# Emerging Roles of Extracellular Vesicle-Mediated Transfer of Mitochondrial and Mitochondrial Components in Cancer

**DOI:** 10.3390/cells15161502

**Published:** 2026-08-20

**Authors:** Yue Gu, Chen Gu, Runfang Pan, Baonian Liu

**Affiliations:** Department of Anatomy, School of Integrative Medicine, Shanghai University of Traditional Chinese Medicine, Shanghai 201203, China

**Keywords:** extracellular vesicles, mitochondrial transfer, mtDNA, cancer

## Abstract

Extracellular vesicles (EVs) are crucial mediators of intercellular communication in the tumor microenvironment (TME) which facilitate the transfer of bioactive molecules including functional mitochondria and their integral components. This review summarizes the emerging role of EV-mediated mitochondrial transfer in cancer progression. We delineate the mechanisms governing the packaging of mitochondria and their constituents into EVs and subsequently highlight their multifaceted functions across various malignancies, including breast cancer, prostate cancer, blood malignancies, head and neck squamous cell carcinoma, digestive system cancers, etc. Mitochondrial cargo, such as intact mitochondria, mitochondrial DNA (mtDNA), and RNA (mtRNA), are shown to reconfigure metabolism, enhance bioenergetics, promote proliferation and invasion, induce drug resistance, and remodel TME by suppressing antitumor immunity. While previous reviews have predominantly focused on the role of mitochondrial transfer in individual cancers or specific systemic diseases, we made a comprehensive overview encompassing diverse cancer types. These findings suggest that EV-mediated mitochondrial cargo transfer represents a biological intercellular communication mechanism with implications for tumor progression and therapeutic resistance. It is worth noting that we also apply standardized evidence-grading frameworks (C1–C4) across cancer types to provide a critical assessment of the current evidence and identify key methodological gaps that must be addressed in future studies. Collectively, this review underscores the significance of EV-mediated mitochondrial transfer as an important biological process in cancer, presenting it as a promising frontier for novel diagnostic and therapeutic interventions.

## 1. Introduction

Globally, malignancies constitute a profound health crisis, consistently exhibiting high incidence and mortality rates that impose substantial social and economic burdens. GLOBOCAN data released in 2022 by the International Agency for Research on Cancer (IARC) show that new cancer cases worldwide totaled nearly 20 million, with associated fatalities reaching 9.7 million. Furthermore, global cancer incidence is projected to surge to 35 million by 2050 [1]. Mechanistically, oncogenesis and tumor progression are multistage processes governed by genetic mutations, epigenetic alterations, dysregulated signaling cascades, and TME remodeling. The rapid proliferation of malignant cells is heavily dependent upon aerobic glycolysis (the Warburg effect) [2], as well as cellular respiration and other mitochondrial functions [3]. In addition, the utilization and metabolism of nutrients provide biomarkers related to disease progression [4], affect immunity and differentiation, and promote or prevent anticancer responses [5]. Fortunately, significant advances have been made in current diagnostic and therapeutic technologies. For example, combination therapies incorporating notch inhibitors and gamma-secretase inhibitors [6] specifically target cancer stem cells to mitigate drug resistance and disease relapse [7]. Percutaneously delivered, minimally invasive, image-guided locoregional interventions have also become definitive therapeutic options for patients with early-stage malignancies or oligometastatic disease in diverse anatomical sites [8]. Recently, comprehensive genomic profiling tests have started in clinical trials for advanced or metastatic solid tumor [9]. Substantial progress has been achieved in HER2-targeted therapy development, encompassing monoclonal antibodies, tyrosine kinase inhibitors, antibody–drug conjugates (ADCs), and novel bispecific agents [10]. The cellular immunotherapy landscape for central nervous system tumors may be transformed by recent developments in chimeric antigen receptor T-cell engineering, combinatorial treatment regimens, and CNS-targeted approaches [11]. Additionally, other treating strategies include surgery [12], radiotherapy [13], chemotherapy [14], and immunotherapy [15]. However, tumor recurrence [16], metastasis [17], and drug resistance [18] remain major obstacles in clinical treatment. Therefore, deep exploration of mechanisms underlying tumor occurrence and progression is of great importance for developing effective treating strategies.

Mitochondria exhibit highly dynamic morphologies, ranging from spherical to elongated architectures, and are encapsulated by a double membrane [19]. The inner membrane invaginates to form cristae, which compartmentalize the matrix containing mtDNA, ribosomes and metabolic enzymes [20]. Beyond previously documented mitochondria-nuclear envelope contacts implicated in prosurvival signaling in yeast and neoplastic models, recent studies demonstrate a direct structural interplay between mitochondria and the nuclear pore complex. Specifically, the outer mitochondrial membrane protein VDAC1 has been identified as the primary binding partner for the filamentous nucleoporin RANBP2 [21]. Within eukaryotic systems, mitochondria act as central metabolic hubs essential for cellular bioenergetics, signaling cascades, and metabolic homeostasis [22]. Through oxidative phosphorylation (OXPHOS), these organelles supply the ATP required to sustain physiological and oncogenic cellular activities. Furthermore, mitochondria are intimately involved in orchestrating multiple pivotal cellular mechanisms, including metabolic reprogramming, reactive oxygen species (ROS) generation, and apoptotic pathways [23]. As the center of oxidative metabolism, mitochondria harbor their own autonomous genetic material. Accumulating evidence has established that mitochondrial dysfunction is related to large amounts of human diseases, including age-related endothelial dysfunction, cardiovascular disease [24] and chronic obstructive pulmonary disease [25]. Moreover, mitochondria also exacerbate pathogenesis of human neurodegenerative disorders by activating cell death and inflammatory pathways [26]. Consequently, mitochondria occupy a pivotal position in maintaining overall cellular physiology. Dynamic intercellular mitochondrial exchange is pivotal to cellular and tissue physiology, as it restores mitochondrial metabolism, ensures the quality control of donor cells, and promotes tissue homeostasis and remodeling [27]. Concurrently, a recent study has demonstrated a mitochondrial transplantation approach based on encapsulating these organelles within erythrocyte plasma membrane-derived vesicles, which effectively rescues the bioenergetic and biochemical defects associated with mitochondrial diseases [28].

Extracellular vesicles (EVs) [29] are cell-derived nanovesicles delineated by a lipid bilayer that exhibit heterogeneous sizes, encompassing subpopulations such as microvesicles, microparticles, and ectosomes [30]. Their biogenesis occurs via two primary mechanisms: the endosomal pathway, or direct vesicle formation at the plasma membrane. They have the ability to change secretion and metabolism by transporting bioactive cargo between target cells or organs. Recently, EV-mediated delivery of mitochondria or their components has drawn considerable research interest. Pathogenic or therapeutic development for various mitochondria-related diseases has sprung up. Recent studies have demonstrated mitochondrial EVs can act as biological markers in innovatively treating pulmonary diseases [31], induce liver injury by triggering cytokine release and pro-inflammatory immune activation, exacerbate myocardial tissue damage, culminating in cardiovascular diseases [32], and influence tumor progression, the tumor microenvironment [33], and resistance. For instance, in microglia, glycoprotein non-metastatic melanoma protein B (GPNMB) shuttles via EVs to astrocytes, where it assembles on mitochondria with Parkin/Nix and drives the release of functional mitochondria-containing vesicles, thereby bolstering astrocytic function and alleviating the cognitive decline and pathological features associated with Alzheimer’s disease [34]. Conversely, in Osteoarthritis, mesenchymal stem cells derived from the infrapatellar fat pad (IPFP-MSCs) generate mitochondria-derived vesicles (MDVs) that directly fuse with chondrocytes to deliver exogenous mtDNA; this delivery inhibits matrix synthesis, induces mitochondrial dysfunction, and activates pro-inflammatory signaling cascades [35].

In recent years, EVs and EV-derived cargos have attracted continuous attention and the study of mitochondria transfer has become a hot topic in the field of cancer research. However, most existing reviews tend to focus on isolated tumor types (e.g., lung, breast, or liver cancer individually), there are still some questions remain unsolved. For example, the therapeutic mechanism, detailed cargos, and effects of mitochondrial EVs in different diseases need to be elucidated. In this review, rather than simply enumerating the types of mitochondrial cargo detected in EVs, we systematically evaluate the evidence base using a predefined hierarchy that distinguishes detection, localization, functional effects, and causal validation. This framework allows us to identify where the field stands across different cancer types and to highlight specific methodological gaps that need to be addressed, helping us advance from descriptive observations to mechanistic understanding. Given the rapidly expanding and fragmented literature in this field, we believe that a timely synthesis of the available evidence offers valuable guidance for future research directions, especially the mechanistic insights and therapeutic implications. We aim to provide a pan-cancer landscape, integrating the commonalities and divergences of mitochondrial transfer mechanisms among different malignancies.

According to MISEV2023 guidelines, it is essential to distinguish several distinct entities that are discussed throughout this review. EVs are defined as particles released from cells, delimited by a lipid bilayer, and cannot replicate on their own. Within this broad category, EV subtypes may be further described based on size, density, molecular composition, or cellular origin. It is worth mentioning that mitochondrial-derived vesicles (MDVs) are not synonymous with EVs. MDVs are vesicles budded from the mitochondrial surface that primarily function in intracellular quality control pathways, targeting lysosomes or peroxisomes for degradation, although they may ultimately be released extracellularly. So, in this review, we clearly delineate EV-mediated shuttling (includingexosomes, microvesicles, and oncosomes), non-EV intercellular routes (TNTs, gap junctions, and free mitochondria uptake), and specialized EV subtypes, including exophers and migrasomes.

We conducted a narrative literature review based on a structured search of PubMed and Web of Science databases up to June 2026. The search terms included combinations of the following keywords: “mitochondrial transfer”, “intercellular mitochondrial trafficking”, “extracellular vesicles”, “cancer”, “tumor microenvironment”, and “therapeutic implications”. References cited in relevant original articles and previous reviews were also manually screened to ensure comprehensive coverage. Only English-language articles were included in this review. First, we will briefly introduce the relationship between mitochondria, extracellular vesicles and cancer diseases. Subsequently, we will sort out how these components enter EVs, and focus on their main effects and different pathways resulting from transferring in cancers such as breast cancer, prostate cancer, lung cancer and blood malignancies, which may provide insights into further clinical practice. In this review, EV-mediated transfer refers exclusively to the shuttling of mitochondrial components via exosomes, microvesicles, and oncosomes. However, to provide a comprehensive overview of the intercellular mitochondrial landscape, we also briefly discuss alternative non-EV routes, including tunneling nanotubes (TNTs), gap junctions, and uptake of free mitochondria. These non-EV pathways are clearly distinguished from EV-mediated processes throughout the text.

## 2. Mitochondria and Mitochondrial Components in Cancers

Beyond energy production and macromolecular synthesis, mitochondria exert a complex, paradoxical influence on oncogenesis [36]. Elevated mitochondrial metabolic flexibility enables tumor cells to sustain rapid proliferation and survive microenvironmental stressors, such as hypoxia and nutrient limitation. For example, intratumoral conventional type 1 dendritic cells (cDC1s) adopt heterogeneous mitochondrial states, wherein OPA1-modulated mitochondrial bioenergetics and redox balance govern their antitumor responses [37]. Additionally, horizontal mitochondrial transfer between glioblastoma cells and their microenvironment, particularly involving astrocytes, promotes tumor progression, metabolic reprogramming, and therapeutic resistance [38]. On the other hand, although mitochondria are utilized by eukaryotic cells to sense cellular damage and elicit effective immune responses [39], the dysregulation of mitochondria-mediated immune escape pathways represents a critical mechanism through which tumor cells evade programmed cell death. Within TME, mitochondria exert potent regulatory influences on immune responses via the modulation of T-cell survival and function, macrophage polarization, NK-cell cytotoxicity, and neutrophil activation [40]. In nutrient-scarce microenvironments, tumor-associated macrophages rely on TRAP1-regulated mitochondrial activity to maintain an immunosuppressive phenotype [41]. Furthermore, malignant cells can physically acquire mitochondria from neighboring immune cells. This mitochondrial depletion impairs the antigen-presentation and co-stimulatory capacity of immune populations, dampening downstream NK-cell and CD8^+^ T-lymphocyte activation. Once inside cancer cells, these acquired organelles fuse with the host mitochondrial network, releasing cytosolic mtDNA to trigger cGAS/STING signaling and drive type I interferon-dependent immune evasion [42]. In addition, mitochondrial-derived factors, including mtDNA, mtROS, and specific metabolic intermediates, act as direct signaling molecules that regulate inflammatory responses, immune evasion, and metastatic dissemination. For instance, VDAC is intimately linked to both OXPHOS and glycolysis [43]. Research has shown that TRIM21, a protein endogenous to tumor cells, restrains mtDNA from releasing and inhibits antitumor responses via degrading VDAC2 in nasopharyngeal carcinoma [44]. Furthermore, various cancer cells have been shown to donate mitochondria to fibroblasts in co-culture models and xenograft tumors through the mitochondrial trafficking protein MIRO2. This intercellular transfer drives a protumorigenic cancer-associated fibroblast (CAF) phenotype by reprogramming fibroblast metabolism, upregulating CAF markers, and inducing the secretion of a protumorigenic secretome and matrisome [45]. Therefore, elucidating the roles and specific mechanisms of mitochondria during tumor progression will not only enhance our understanding of cancer biology, but may also provide emerging perspectives for developing novel anticancer therapies.

Mitochondrial damage is driven by multiple mechanisms, including the disruption of mitochondrial membrane potential by uncoupling agents, respiratory chain inhibition leading to ROS accumulation, mitophagy induction by iron chelators via iron depletion, the blockade of autophagic flux by mitophagy inhibitors, and oxidative stress induced by non-targeted ROS generation [46]. Mitochondria frequently undergo morphological and spatial remodeling in response to cellular stress and energy demands [47]. Therefore, the intercellular transfer of dysfunctional mitochondria is pivotal for maintaining tissue homeostasis, exerting either anticancer or carcinogenic effects. Accumulating evidence reveals that cellular damage can be repaired by integrating exogenous mitochondria into the host mitochondrial network, thereby driving cancer cell expansion and resistance to therapy [48]. For instance, the donation of damaged mitochondria to mesenchymal stem cells (MSCs) shows attenuated ROS levels and promotes survival. In alveolar type 2 epithelial cells, the secretion of damaged mitochondrial components via small EVs relies on a GPRC5A–MIRO2-dependent pathway, operating as an extracellular mitochondrial quality-control mechanism that preserves epithelial homeostasis and alleviates lung injury [49]. Extracellular mitochondrial clearance also intersects with oncogenesis; for instance, purging mtDNA limits tumor development [3], whereas downregulation of DRP1, a key regulator of mitochondrial fission, impairs the survival capacity of breast cancer brain metastases [50]. Together, these pathways illustrate that cells actively discharge mitochondria and their constituents into the extracellular space under both homeostatic and pathological states, facilitating functional transfer between donor and recipient cells.

## 3. Extracellular Vesicle in Cancers

**Figure 1:**
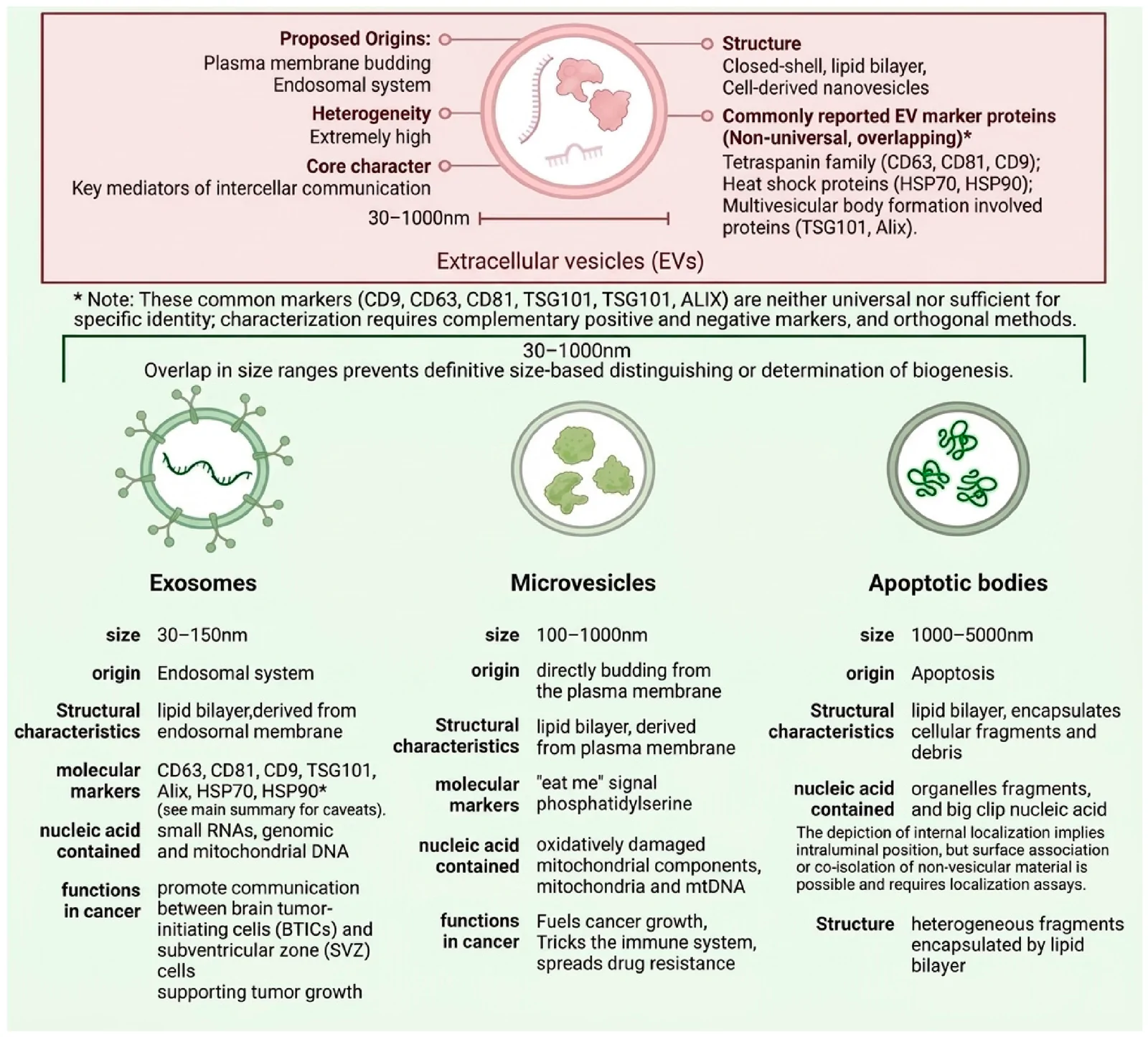
Major EV categories and commonly reported characteristics. Extracellular vesicles are lipid bilayer membrane structures released by cells. Markers listed such as CD9, CD63, CD81, TSG101, and ALIX represent commonly reported and enriched EV proteins rather than universal or exclusive lineage markers, because rigorous EV characterization requires combining positive markers with negative/contaminant controls (e.g., non-EV proteins) and complementary orthogonal physical and biochemical methods (MISEV guidelines). Although distinct operational size ranges are depicted, a significant size overlap exists across subtypes. Physical size alone cannot definitively resolve biogenic origin or distinguish subtype identity. Depicted internal nucleic acids (small RNAs, genomic DNA, and mtDNA) and proteins represent total associated cargo. Intraluminal localization cannot be assumed by default, as molecules may reside on the outer membrane surface or co-isolate as nonvesicular ribonucleoprotein complexes. Verification of true intraluminal encapsulation requires protection assays (e.g., enzymatic digestion with RNase/Proteinase K) combined with membrane permeabilization.

Encapsulated by lipid bilayers, EVs are secreted across nearly all cell lineages, exhibiting a broad size spectrum from 30 to 1000 nanometers [51]. Long discarded as simple cellular waste conduits, EVs are now recognized as essential mediators of intercellular communication [52]. Through the transport of bioactive molecular cargo, including RNA, lipids, proteins, and metabolic intermediates [51,53,54], EVs regulate a wide range of physiological functions and disease states. For instance, human adipose-derived mesenchymal stem cell exosomes shuttle tumor-suppressive miRNAs into ovarian cancer cells, triggering S-phase cell-cycle arrest, suppressing proliferation and colony-forming capacity, and driving mitochondria-dependent apoptosis via BAX/BCL2/CASP3 pathway activation [55]. Similarly, fatty liver-derived exosomes carrying tRNA methyltransferase 10 homolog C (TRMT10C) selectively accumulate in mammary adipocytes; this vesicular enrichment within the mammary fat pad fosters a protumorigenic breast microenvironment [56]. EVs exhibit profound heterogeneity and are classified into distinct subpopulations based on their size, molecular cargo, and biogenetic pathways (Figure 1), chiefly comprising exosomes, microvesicles and apoptotic bodies [57]. For example, exosomes have been demonstrated to augment thyroid cancer growth via induction of glycolysis [58]. Additionally, recent evidence indicates that tumor-derived blebbisomes, which can reach up to 20 µm in diameter, carry numerous inhibitory immune checkpoint proteins, e.g., PD-L1, B7-H3, VISTA, PVR, PD-L2, and HLA-E, and function as autonomous communication platforms that integrate extracellular signals and mount appropriate responses [59]. As research advances, EVs have emerged as novel targets and tools for disease diagnosis, prognostic assessment, and therapeutic intervention, demonstrating particularly significant progress particularly in the field of oncology.

EVs express specific surface marker molecules that not only facilitate their identification and isolation, thus conditioning tumor growth, invasion, and metastasis [60,61]. Canonical EV marker proteins include members of the tetraspanin family (CD63, CD81, CD9), heat shock proteins (HSP70 and HSP90), and multivesicular body-associated proteins (TSG101 and ALIX) [62]. Within the head and neck squamous cell carcinoma (HNSCC) microenvironment, tumor-derived exosomal PA28γ triggers T-cell exhaustion by diminishing the cytotoxic capacity of these cells, thereby fostering malignant progression through the PA28γ/BCLAF1/CD25-LAG-3 axis [63]. However, the expression profiles of EV surface markers exhibit significant heterogeneity, which is heavily dictated by their cellular origin and underlying physiological or pathological status. For instance, tumor-derived EVs frequently present elevated levels of immune checkpoint proteins like PD-L1 and HLA-G [64], whereas stem cell-derived EVs may express specific differentiation antigens.

Concurrently, EVs encapsulate a diverse repertoire of biomolecules, including proteins, DNA, mRNA, miRNA, lncRNA, lipids, and metabolites. Not only do these cargos reflect the pathophysiological state of their parent cells, but they can also exert functional effects within recipient cells. EVs can drive tumor cell proliferation via the transfer of growth factor receptors or oncogenic factors. For example, In glioblastoma, TEVs mediate cross-talk between brain tumor-initiating cells and subventricular zone niches to sustain growth [65]. Furthermore, TEVs carrying pro-angiogenic factors, such as VEGF and FGF, stimulate neovascularization to satisfy the metabolic demands of expanding tumors [57]. Prostate cancer cells secrete exosomal PD-1 to activate myeloid-derived suppressor cells (MDSCs) via JAK/STAT3 signaling, which in turn restricts CD8^+^ T-cell infiltration within the TME to facilitate immune evasion [66]. Similarly, red blood cell-derived exosomal miR-93-5p targets PTEN in recipient cancer cells, enhancing proliferation, migration, and invasive capacity [67]. Beyond direct effects on tumor cells, EVs reconfigure the surrounding microenvironment by co-opting stromal and immune compartments. For example, EVs isolated from bone marrow aspirates of multiple myeloma patients exhibit high levels of PD-L1, HLA-G, and PD-1, suppressing T-cell activation and cytokine production to enforce an immunosuppressive milieu [64]. Moreover, TEVs presenting pro-apoptotic factors such as Serpin B9 directly trigger apoptosis in chimeric antigen receptor (CAR) T-cells, blunting immunotherapeutic efficacy [62]. Conversely, non-malignant extracellular vesicles can exert antitumor effects; skeletal muscle-derived small EVs (C2C12-EVs) inhibit proliferation, induce S-phase arrest, impede cell motility, and trigger intrinsic mitochondrial apoptosis in murine lung carcinoma models [68]. Additionally, EV-mediated efflux or sequestration of chemotherapeutic agents directly drives acquired multidrug resistance [69].

Harnessing these mechanisms has enabled novel cell-free therapeutic strategies. For instance, engineered exosomes derived from B7-H3-targeted CAR-T cells loaded with miR-145 (termed exo-CT-145) block tumor initiation and progression across multiple targets in solid tumor models [70]. Similarly, irradiated tumor cell-derived microparticles (RT-MPs) exhibit intrinsic tumor-homing capacity and innate immune-activating properties; loading RT-MPs with chemotherapeutic payloads (methotrexate, monomethyl auristatin E, or doxorubicin) yields integrated chemoradiotherapeutic platforms that suppress tumor growth, extend host survival, and maintain favorable systemic biosafety [71]. Furthermore, in a triple-negative breast cancer (TNBC) orthotopic model, siT/MOF@EVs effectively overcame tumor heterogeneity, suppressed tumor growth and dissemination, and induced a protective immune memory response without inducing systemic toxicity [72]. Similarly, Sor@AKAExo—a bioinspired platform utilizing Anoectochilus roxburghii-derived exosomes to co-deliver endogenous miRNAs and the ferroptosis inducer sorafenib—synergistically induces ferroptosis and TNBC apoptosis through GPX4 suppression, lipid peroxidation, mitochondrial dysfunction, and caspase-3 activation [73]. In another approach, a Golgi apparatus-targeted, aggregation-induced emission photosensitizer (TCN) was rationally engineered and encapsulated within biomimetic hybrid EVs functionalized with M1-type macrophage membranes (EM@TCN); this platform functions as a pyroptosis inducer, thereby remodeling the tumor microenvironment and potentiating immunogenicity [74]. Finally, bioinformatics screening identified HSPD1 overexpression in prostate cancer, which prompted the development of siRNA-loaded EVs (siRNA@EVs) targeting HSP60 (encoded by *HSPD1*), which achieved potent gene silencing and suppressed PCa proliferation and metastasis. [75].

## 4. Mechanism of Mitochondrial Intercellular Transfer

To date, several distinct mechanisms of intercellular mitochondrial trafficking have been elucidated. It is noteworthy that non-EV routes, including TNTs, tumor microtubes (TMs), gap junction intercellular communication (GJIC) and cell fusion, have also been reported to transfer mitochondrial components [76]. Although these are beyond the primary EV scope of this review, they are briefly summarized below for comprehensive background.

**Figure 2:**
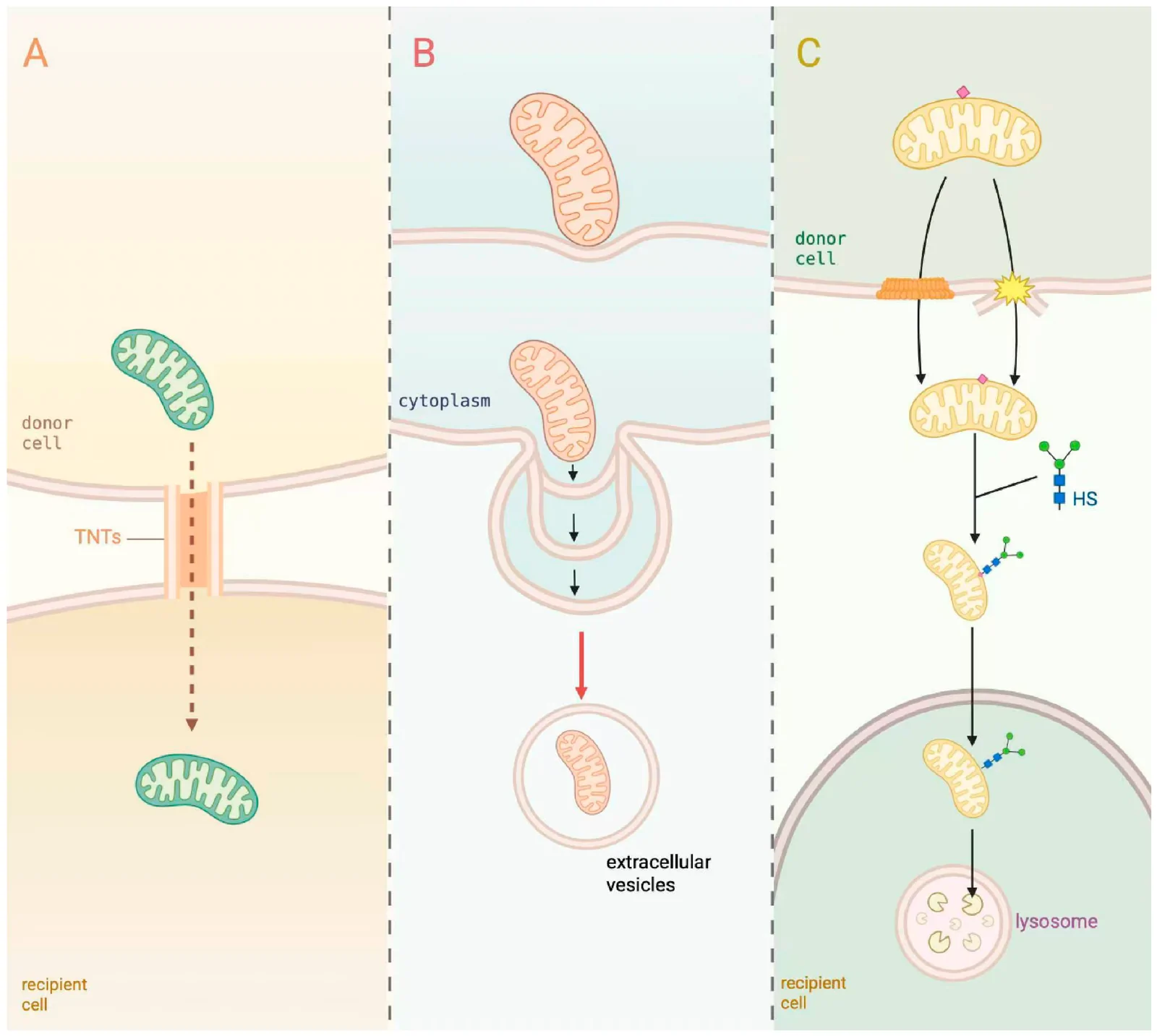
Overview of intercellular mitochondrial component transfer mechanisms. There are many ways for intercellular transport within mitochondria, including EV-mediated routes and non-EV intercellular routes. These mechanisms can be mainly concluded as the following three kinds. (**A**) Non-EV intercellular transfer routes: this includes TNTs, which form direct membranous connections between physically connected cells, and the brown dashed arrow indicates direct intercellular translocation of mitochondria through TNTs; (**B**) EV-dependent release routes: mitochondrial components are packaged into EVs (exosomes, microvesicles, or oncosomes) and released into the extracellular space, and solid arrows depict sequential stages of membrane invagination, budding, and vesicle release; (**C**) recipient-cell uptake mechanisms: once free mitochondria reach the extracellular space, recipient cells internalize them via endocytosis or phagocytosis, and solid black arrows outline the pathway of surface recognition, endocytic internalization, and subsequent lysosomal targeting. These uptake steps are mechanistically distinct from the release/transfer processes shown in panels (**A**,**B**).

Findings from recent years indicate that mitochondria transfer mechanisms can be summarized into three groups (Figure 2): (A) formation of transient cellular connections or signals, (B) intercellularly delivery of mitochondria in EVs, and (C) unbound mitochondria release being taken up by recipient cells [77].

The first mechanism is direct cell–cell contact-mediated transfer, primarily recognized as TNTs and/or Connexin-43-mediated GJICs. With diameters ranging between 50 and 200 nm in diameter, TNTs are long, slender membranous tubules containing cytoskeletal filaments, predominantly F-actin and microtubules [78], whose extension facilitates the movement of organelles across cells that are physically connected [79]. Specifically, a recent study demonstrated that breast cancer cells exposed to neurons acquire neuron-derived mitochondria primarily through tunneling nanotube-like structures, thereby confirming active mitochondrial transfer within nerve-cancer co-cultures [80]. Similarly, glioblastoma cells acquire mitochondria from astrocytes through GAP43-dependent intercellular bridges, thereby augmenting oxidative metabolism and self-renewal capacity [81]. Secondly, mitochondria or mitochondrial-derived cargo are contained in vesicles decorated with numerous marker proteins. Among them, EVs account for a large proportion in intercellular horizontal transfer of mitochondria. In 2016, Daniel Torralba [79] concluded that many mitochondrial components had been detected in EVs, but how these mitochondrial components are loaded in EVs remains unknown. Exosomes hold mostly small RNAs, as well as genomic and mitochondrial DNA; small EVs often contain oxidatively damaged mitochondrial components; larger EVs contain intact mitochondria and mtDNA. For instance, mesenchymal stem cells (MSCs) produce arrest in domain-containing protein1-mediated microvesicles to target depolarized mitochondria toward the cell surface, and the EVs are engulfed and reutilized by the fusion of macrophages [82]. In white and brown adipocytes, EVs generated via a PINK1-dependent pathway carry CD63, CD9, and CD81 on their surface [83]. Leydig cells release EVs carrying defective mitochondria, which are subsequently cleared by CD206^hi^ testicular macrophage (tMac) in a TREM2-dependent manner. Notably, TREM2 deficiency in tMacs impairs testosterone synthesis; conversely, Leydig cells acquire functional mitochondria-loaded vesicles from MHCII^hi^tMacs through ITGβ1-VCAM1 interaction [84]. Cardiomyocytes release large, dysfunctional mitochondria within exophers decorated with LC3. These structures bud off the cell surface in a Rab7-GDP-dependent fashion [85,86].

Additionally, cells release whole free mitochondria and recipient cells take them up through a phagocytic mechanism mediated by heparan sulfate (HS) [87]. Then, they are degraded by the lysosome. Despite all the mechanisms mentioned, a number of other intercellular transfer ways have been observed, such as cell fusion and gap junctions. For instance, mitochondria components are transported from bone marrow mesenchymal stem cells to motor neurons by way of gap junctions [79], whereas conventional gap junctions, due to their narrow pore size (1.5–2 nm), are structurally incapable of accommodating intact mitochondria. Via complete or partial cellular fusion, cardiomyocytes and stem cells can regain mitochondrial respiratory activity [88]. To date, several critical mechanistic questions remain unresolved: the precise mechanisms governing the release of MDVs and their subsequent interaction with target cells, the downstream events following their cellular uptake, and their ultimate intracellular destination within recipient cells.

## 5. Extracellular Vesicle-Mediated Mitochondrial and Mitochondrial Components Transfer in Cancers

To critically appraise the literature on EV-mediated mitochondrial cargo transfer, we established a standardized evidence hierarchy and applied it across all cancer types discussed in this review. Studies were categorized into four evidence levels (C1–C4). C1 (Detection): Studies detected cargo in EV-enriched preparations, but do not confirm intravesicular localization, contaminant exclusion, or intercellular delivery. C2 (Localization): Intravesicular localization and/or contaminant exclusion demonstrated (e.g., detergent protection, nuclease protection, immunogold, EV positive/negative markers). Both intercellular delivery and cargo localization are sufficient for C2. C3 (Functional Effect): Cargo localization (C2) AND evidence of intercellular delivery AND functional effects in recipient cells (e.g., OCR, ATP, migration, chemoresistance). C4 (Causal Validation): Studies that include C3 criteria AND cargo-specific loss-of-function or rescue experiments (KO, siRNA, pharmacological inhibition, complementation) to establish that the observed phenotype is directly attributable to the transferred mitochondrial cargo. Evidence quality (High, Moderate, or Low) was further assessed based on the rigor of EV purification methods (e.g., density gradient vs. conventional differential ultracentrifugation), the use of appropriate EV positive and negative markers, exclusion of apoptotic bodies and cell-death products, and the completeness of functional validation. Importantly, we emphasize that the detection of mitochondrial components in EV lysates by Western blot, PCR, or MitoTracker staining does not establish intravesicular cargo localization, exclude free mitochondrial or cellular contaminants, demonstrate recipient-cell uptake and intracellular cargo release, or confirm integration into the recipient mitochondrial network and restoration of bioenergetic function for intact mitochondria. These distinctions, which are central to interpreting the evidence base, are explicitly annotated in Table 1, Table 2 and Table 3 and are addressed in the cancer-specific discussions below.

### 5.1. Breast Cancer

**Figure 3:**
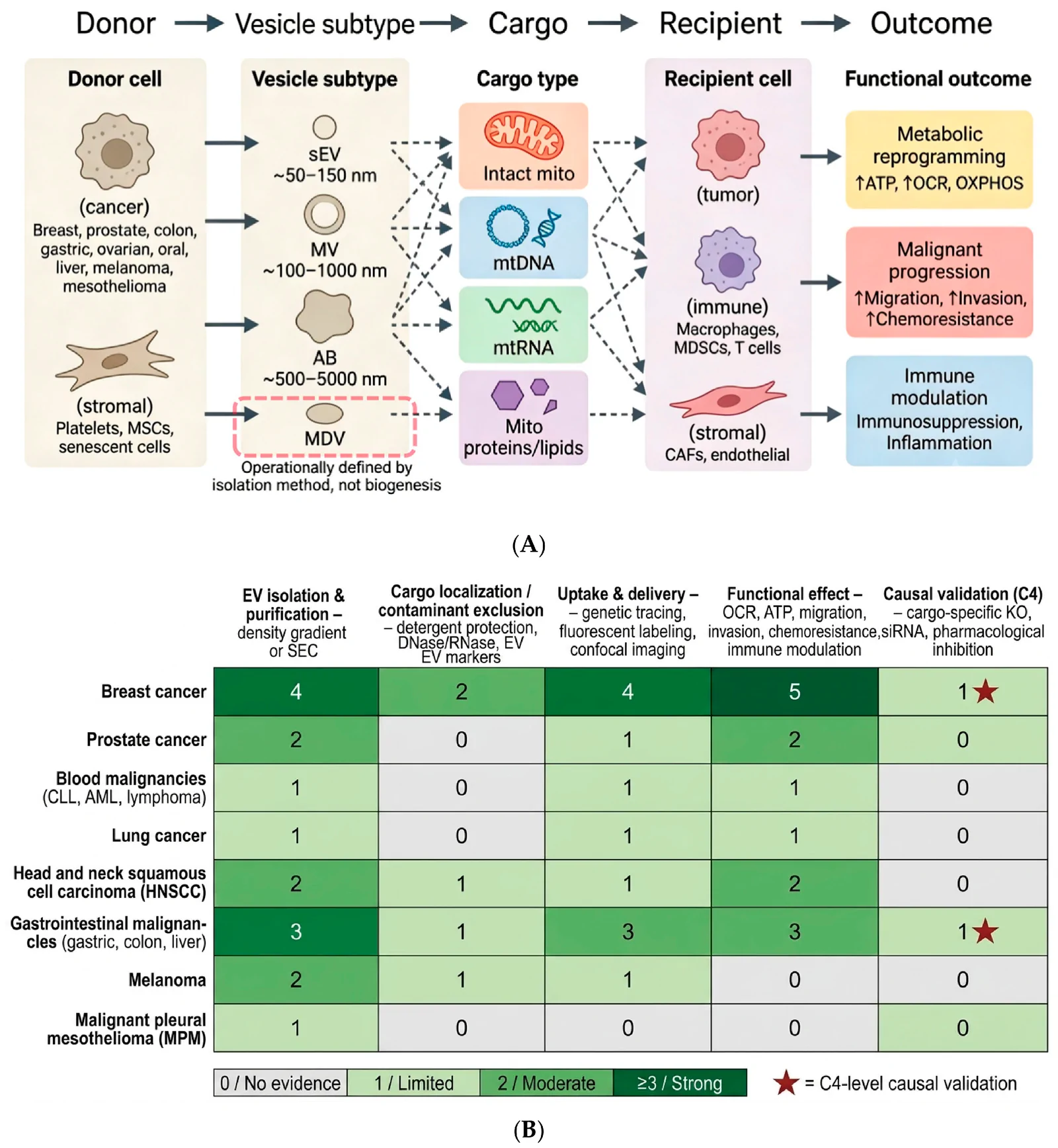
(**A**). Schematic flow of EV-mediated mitochondrial cargo transfer. Donor cells (cancer or stromal) release vesicles (including sEVs, MVs, apoptotic bodies/lipidosomes, and MDVs) carrying mitochondrial cargo (intact mitochondria, mtDNA, mtRNA, or mitochondrial proteins/lipids). Arrows indicate the sequential flow of mitochondrial component transfer from donor cancer cells to recipient cells via distinct vesicle subtypes, ultimately leading to the indicated functional outcomes in the recipient cells. MDVs are included for completeness as they can be released independently or incorporated into EVs. However, they are not classical EVs and originate from mitochondria via a distinct budding process. These vesicles are then taken up by recipient cells (tumor, immune, or stromal), leading to functional outcomes including metabolic reprogramming, malignant progression, or immune modulation. Dashed arrows indicate pathways observed in specific models but not universally established. Vesicle subtypes are defined by operational isolation methods, not by biogenesis. See Table 1, Table 2 and Table 3 for study-specific details and evidence grading. (**B**). Evidence matrix for EV-mediated mitochondrial cargo transfer across cancer types. This matrix visualizes the evidence strength across eight cancer types and five evidence criteria based on the studies reviewed (Table 1, Table 2 and Table 3). Color intensity reflects the number of independent studies meeting each criterion: dark green, ≥3 studies; medium green, 2 studies; light green, 1 study; gray, no studies. Numbers inside cells indicate the count of studies satisfying the criterion. ★ marks the presence of C4-level causal validation (cargo-specific loss-of-function/rescue experiments). Diagnostic studies (C0) listed in Table 3 were excluded from this matrix to maintain focus on functional evidence; they are discussed separately in the main text. Absence of evidence does not imply absence of effect. Detailed study references are provided in Table 1, Table 2 and Table 3.

Breast cancer represents the predominant female malignancy globally, accounting for approximately 2.5 million new cases and 700,000 deaths annually [89]. Accumulating evidence has demonstrated that EVs mediate the transfer of functional mitochondria and mitochondrial components in breast cancer. Platelets serve as a primary source of intact mitochondria via the release of microvesicles. The platelet-derived microvesicles (PMVs) carries various contents derived from platelets, such as proteins and nucleic acids, both contributing to the process of tumor cell intravasation [90]. Recipient breast cancer cells can internalize exogenous mitochondria to restore impaired mitochondrial function, a process dependent on the upregulation of the platelet activation marker (CD62p) on their plasma membrane [91]. This horizontal transfer subsequently elevates intracellular ATP levels and O_2_ consumption rate (OCR) [92]. Consequently, the recipient cancer cells exhibit enhanced proliferative capacity, increased invasion, and exacerbated malignant phenotypes. Conversely, through direct contact with breast cancer cells, mammary epithelial cells suppress tumoroid formation, a process linked to the heightened transcriptional activity of HSPD1, TP53, TIMM9, TIMM10, SLC25A24, SLC25A4, LRPPRC and BCL2, but make a smaller contribution than TNTs in this context [93]. Furthermore, breast cancer cells themselves can generate EVs, and mitochondrial protein packaging occurs in exosomes produced by cancer-derived cell lines [94]. Cancer cells under metabolic stress release PINK1-dependent, mtDNA-containing EVs, which activate TLR9 in recipient cells and therefore promote pro-invasive endosomal trafficking, driving increased invasiveness and metastasis [95]. Plasma EV RNA profiling has identified altered levels of mitochondrially encoded mRNAs related to oxidative phosphorylation and the electron transport chain, alongside differential expression of miR-200b-5p, miR-218, and other miRNAs, in breast cancer patients compared with healthy controls [96], although the functional implications of these alterations for tumor metastasis and proliferation remain to be established. Among breast cancer subtypes, triple-negative breast cancer (TNBC) stands out as one of the most aggressive with the poorest prognosis following chemotherapy or radiotherapy. EVs originating from TNBC cells are capable of delivering mitochondria to chemosensitive recipient cells, thereby increasing their chemoresistance to ensure survival [97]. Recent research has shown that the removal of damaged mitochondrial components from breast cancer cells can also occur via EVs through the metabolism of ceramide and glycated derivatives along with lysosome-mediated mitochondrial degradation [98]. These findings collectively highlight the significance of intercellular communication in breast cancer, presenting EV transfer as a critical mechanism underpinning tumor metabolism and progression, which could be exploited for future therapeutic interventions (Figure 3, Table 1, Table 2 and Table 3).

Applying our evidence framework to breast cancer studies, only one study ([95]) satisfied causal (C4) validation by performing specific knockdown/rescue experiments to establish PINK1-dependent mtDNA packaging into EVs and downstream TLR9-mediated functional effects. The remaining functional studies ([91]) provide low-to-moderate evidence quality, largely restricted to detection (C1) or qualitative functional observations due to methodological limitations, such as omitting density-gradient purification, detergent protection assays, or controls for free mitochondria and apoptotic bodies. Notably, study [96] represents a diagnostic biomarker study evaluated separately in Table 3 (C0), whereas study [99] characterizes secretory mitophagy rather than canonical EV-mediated intercellular transfer to recipient cancer cells, requiring independent interpretation.

### 5.2. Prostate Cancer

Prostate cancer accounts for substantial male cancer mortality globally. While androgen receptor pathway inhibitors, taxane-based chemotherapy, and radiopharmaceuticals have expanded therapeutic options [100], disease progression remains heavily influenced by cell–cell communication within the TME. A critical mode of communication between prostate cancer cells and stromal cells or neighboring tumor cells within the prostate tumor milieu is to transfer mitochondria via EVs formed with the help of ERM proteins (such as ezrin). This phenomenon illustrates a form of metabolic symbiosis, wherein tumor cells fuel the aggressiveness of their neighboring ones. Apart from transferring mitochondria, these EVs also transport lysosomes, lipids, the endoplasmic reticulum, syndecan-1, sortilin, GLUT1, GLUT4, and the androgen receptor (AR) to supply energy and enhance glycolytic capacity [101]. Radiation stress further amplifies this axis: radiation-induced mitochondrial hydrogen peroxide (H_2_O_2_) triggers the release of EVs enriched in mitochondrial proteins that, upon internalization by recipient prostate cancer cells, enhance radioresistance and post-irradiation survival [102]. Furthermore, senescent prostate cancer cells extrude mtDNA into the cytosol and extracellular space via outer membrane VDAC pores; this EV-packaged mtDNA is subsequently taken up by polymorphonuclear myeloid-derived suppressor cells (PMN-MDSCs), stimulating the cGAS-STING-NF-κB signaling cascade to reinforce immunosuppression [103,104]. This process has been linked to the profound inhibition of T-cell proliferation. Together, these findings highlight the importance of blocking EV-mediated mtDNA release in prostate cancer to improve the efficacy of chemotherapy, suggesting that targeting this pathway may represent a novel therapeutic strategy (Figure 3, Table 1, Table 2 and Table 3).

In prostate cancer, two studies ([102,103]) reported EV-associated mitochondrial components—mitochondrial proteins in radiation-derived EVs and mtDNA in senescent cell-derived EVs, respectively—but did not perform detergent protection or DNase treatment to confirm intravesicular localization. Under our cumulative evidence framework, this study is therefore classified as C2, with functional effects discussed qualitatively. Meanwhile, evidence quality was graded as moderate in both cases, as neither study performed detergent protection or DNase treatment to rigorously confirm intravesicular cargo localization. In study [102], the affinity-based EV isolation kit may co-purify non-EV contaminants, and in study [103], causal validation was inferred via indirect VDAC inhibition rather than mtDNA-specific loss-of-function experiments.

### 5.3. Blood Malignancies

Bone marrow (BM) microenvironmental dysfunction underlies diverse hematologic conditions, ranging from non-malignant cytopenias to overt leukemogenesis [105]. In hematologic malignancies, extracellular vesicles (EVs) act as key signaling vectors that drive tumor cell proliferation and promote immune evasion. For acute myeloid leukemia (AML), its incidence increases sharply with age and displays marked geographic variability [106]. EV-mediated mitochondrial transfer regulates immune interactions and cellular clearance mechanisms. These large EVs exhibit enhanced surface exposure of the “eat me” signal phosphatidylserine (PtdSer) exposure, which can bind with bridging molecules Gas6/Protein S to engage MerTK receptors on immune cell surfaces [107]. Notably, inflammatory cytokines, such as IL-1, IL-6, IL-8, IL-10 and TNF, are elevated in patients with AML. Chronic lymphocytic leukemia (CLL) is the predominant adult leukemia in Western populations, with approximately 24,000 new US cases projected annually alongside a substantial prevalent disease burden [108]. Therapeutic paradigms have shifted from chemoimmunotherapy to targeted Bruton’s tyrosine kinase (BTK) and B-cell lymphoma 2 (BCL2) inhibitors. Nevertheless, CLL remains largely incurable due to persistent immune dysregulation, acquired resistance, and cumulative treatment toxicities [109]. From a mechanistic perspective, EVs are transferred from platelets to CLL cells, promoting proliferation by driving cell-cycle progression into the G2/M phase and inducing resistance to chemotherapeutic compounds like Cytarabine, Venetoclax and Plumbagin [110]. The role of EV transfer in hematological malignancies thus extends from metabolic reprogramming to the orchestration of an immunosuppressive niche, highlighting its utility as a biomarker and its therapeutic potential across different leukemia subtypes (Figure 3, Table 1, Table 2 and Table 3).

Among hematological malignancies, study [110] demonstrated that platelet-derived microparticles (PMPs) deliver functional intact mitochondria to CLL cells, enhancing OXPHOS, proliferation, and chemoresistance (C2, Low–Moderate quality). The major limitation of this study is the lack of density gradient purification to exclude free mitochondria and the absence of classical EV markers beyond CD41. Study [107] examined endothelial-derived large EVs in the bone marrow microenvironment of AML and T-ALL models but focused on vascular damage and immune clearance rather than tumor cell-intrinsic mitochondrial cargo transfer; therefore, [107] was excluded from the tables and is discussed here as a contextual observation on EV biology in the leukemia microenvironment.

### 5.4. Lung Cancer

Lung cancer, accounting for 10–12% of cancer-related emergency department visits and 66% hospitalization, is a highly lethal malignancy [111]. Lung cancer continues to be the primary driver of cancer-related mortality globally, with its burden influenced by shifting risk factors, demographic transitions, and healthcare inequities. Although age-standardized incidence and mortality rates have trended downward over recent decades, the absolute case count has grown, driven by population expansion and an aging demographic [112]. EVs serve as key functional mediators of local progression and systemic dissemination in lung malignancies. Within the primary TME, cancer cells and stromal populations, including cancer-associated fibroblasts (CAFs), tumor-associated macrophages (TAMs), and bone marrow-derived mesenchymal stem cells (BMSCs), secrete EVs that drive metabolic reprogramming across glucose, lipid, and amino acid networks via the delivery of microRNAs, metabolic enzymes, and pro-inflammatory cytokines. This microenvironmental rewiring accelerates glycolytic flux, induces glutamine dependency, confers resistance to ferroptosis, and promotes epithelial–mesenchymal transition (EMT). Systemically, circulating EVs (encompassing 40–150 nm exosomes and 50–1000 nm ectosomes) traverse vascular and tissue barriers through membrane fusion, receptor endocytosis, or surface ligand–receptor engagement. These circulating vesicles transport catabolic factors, including miR-21, IL-6, HSP70/90, TGF-β, and PTHrP to peripheral tissues, triggering adipose tissue browning, systemic lipolysis, skeletal muscle atrophy, mitochondrial breakdown, and cancer cachexia [113]. Non-small cell lung cancer (NSCLC) accounts for approximately 85% of all lung malignancies, and frequently exhibits intrinsic resistance to conventional chemo- and radiotherapy. Zhou et al. [114] demonstrated that tumor-derived EVs activate CAFs, which subsequently transfer mitochondrial DNA (mtDNA) back to neoplastic cells, restoring oxidative phosphorylation and driving metastatic competence. Providing the most comprehensive evidence to date, Takenaga K [115] established bidirectional EV-mediated transfer of mtDNA harboring the metastasis-enhancing ND6 G13997A mutation between highly metastatic and poorly metastatic Lewis lung carcinoma cells. This vesicular transfer extended to surrounding CAFs, macrophages, and T cells, accomplishing functional metabolic rescue in respiration-deficient ρ^0^ cells (C3, Moderate–High quality). However, key methodological limitations include the lack of detergent-protection assays to confirm true intravesicular encapsulation of mtDNA and an overreliance on broad pharmacological EV inhibitors (GW4869 and tipifarnib) rather than mtDNA-specific genetic ablation. Furthermore, while vesicular transfer of mitochondrial components is well documented, direct evidence demonstrating functional integration and metabolic rescue in recipient tissues, particularly within the context of lung cancer-associated cachexia, remains limited. Addressing these mechanistic gaps is essential to identify actionable pharmacological targets and advance precision therapeutic strategies (Figure 3, Table 1, Table 2 and Table 3).

### 5.5. Head and Neck Squamous Cell Carcinoma (HNSCC)

Arising from the mucosal lining of the upper aerodigestive tract, HNSCC currently ranks as the seventh most common malignancy worldwide [116]. HNSCC often thrives within metabolically challenging microenvironments, where EV-mediated communication plays a crucial adaptive role. In FaDu HNSCC cells, glutamine and serum starvation induced mitochondrial network fragmentation, accompanied by increased TSG101 association, suggesting the potential sorting of mitochondrial cargo into EVs and leading to enhanced abundance of mtRNAs in released EVs, alongside alterations in the EV-associated cytokine secretome including IL-6, IL-13, and CCL5. But the downstream consequences of these EV cargo changes, particularly regarding tumor proliferation, migration, invasion, or metastasis, remain to be established [117]. Specifically, within recipient bone marrow-derived macrophages under hypoxic conditions, hypoxia-inducible factor-1 is upregulated, transcriptionally activating the hepatocyte growth factor-regulated tyrosine kinase substrate to facilitate the assembly of endosomal sorting complexes required for transport (ESCRT). This process gives rise to increased numbers of multivesicular bodies and polarizes macrophages toward a tumor-promoting phenotype [118]. This cascade demonstrates how metabolic stress in HNSCC orchestrates a protumorigenic microenvironment via EV-based mitochondrial component transfer, suggesting that targeting these stress-responsive pathways could disrupt a key survival mechanism. While both studies provide valuable mechanistic insights, study [118] lacks individual mitochondrial protein-specific loss-of-function experiments, and study [117] is purely descriptive without recipient cell functional validation (Figure 3, Table 1, Table 2 and Table 3).

### 5.6. Cancers of the Digestive System

Gastrointestinal tumors utilize EV-mediated mitochondrial transfer to reprogram local metabolism and drive disease progression. In the hepatic niche, liver cancer cells release mitochondrial-derived vesicles (MDVs) to the surrounding stroma, delivering mitochondrial DNA (mtDNA) that functions as an inflammatory damage-associated molecular pattern (DAMP) to promote hepatocellular carcinoma (HCC) progression, immune evasion, and metastasis [119]. HCC accounts for approximately 90% of primary liver malignancies [120] and is characterized by sustained proliferative signaling, angiogenesis, vascular co-option, and immune escape [121]. Furthermore, Liu et al. [122] demonstrated that HSP90 inhibition stimulates MDV formation and the subsequent EV-mediated release of mitochondrial components, accelerating angiogenesis and metastasis (C3, Moderate–High quality evidence); however, the structural and biogenetic distinctions between MDVs and classical EVs remain incompletely resolved.

In the colonic microenvironment, colonic epithelial cells (CECs) internalize mtDNA from adjacent carcinoma cells. This stimulates the mitochondrial respiratory chain and promotes the ROS-dependent nuclear translocation of RelA, which in turn modulates TGF1 expression at the transcriptional level and drives tumor progression via the TGF/Smad axis [123], which achieved the highest evidence level in this review (C4, High quality) with rigorous validation including DNase treatment, long-range PCR, TFAM knockdown, and Rel A ChIP. Accumulating evidence indicates how distinct exosome populations affect gastric cancer (GC), spanning its initial development, subsequent progression, and eventual metastasis, by mediating intercellular communication networks between the tumor and its surrounding stroma [124]. In response to oxaliplatin, GC cells actively deposit ECM within the TME. The biomechanical signals conveyed by this ECM promote mitochondrial transport from MSCs to GC cells, with microvesicles (MVs) serving as the vehicle [125], but this study lacked detergent protection to distinguish vesicle-encapsulated mitochondria from free mitochondria.

Pancreatic carcinoma ranks among the most lethal malignancies [126]. Among these, pancreatic ductal adenocarcinoma (PDAC) represents an exceptionally aggressive neoplasm characterized by a dismal prognosis that is largely attributable to late-stage diagnosis and therapeutic resistance. Furthermore, the global incidence of this disease has been steadily increasing [127]. Because patients predominantly present with advanced-stage disease at the time of diagnosis, contributing to a high mortality rate, the development of rapid and non-invasive screening modalities is imperative. The clinical relevance of EV-associated mitochondrial components in pancreatic cancer has been explored through three distinct lines of investigation with fundamentally different evidence levels. First, Rasuleva et al. [128] identified the ratio of mtDNA to nuclear DNA within circulating tumor-derived EVs as a non-invasive diagnostic biomarker for pancreatic cancer. Second, in bioengineered therapeutic delivery systems, dendritic cell-derived MVs loaded with tumor lysate-pulsed cationic nanoparticles become enriched with mtDNA. This cargo activates the cGAS-STING pathway in recipient dendritic cells, enhancing antitumor immunity and suppressing PDAC and lung tumor growth in vivo [129]. Notably, this represents an exogenous translational application rather than endogenous EV-mediated transfer. Third, Vikramdeo et al. [130] demonstrated that circulating EVs from PDAC patients are enriched in mtDNA and cardiolipin, harboring specific mtDNA mutations with diagnostic utility, though their functional impact remains uncharacterized. Collectively, EV-mediated mitochondrial transfer represents a conserved mechanism across gastrointestinal malignancies that drives metastasis and stromal education, offering novel therapeutic targets in the vesicular trafficking cascade (Figure 3, Table 1, Table 2 and Table 3).

### 5.7. Other Tumors

EV-mediated mitochondrial transfer operates across a broad spectrum of solid tumors, with distinct mechanisms tailored to specific microenvironmental pressures. In ovarian cancer, the late endocytic GTPase RAB7 regulates the trafficking of MDVs to late endosomes for lysosomal degradation. Under stress, cells upregulate the EV-mediated secretion of RAB7, which facilitates cisplatin efflux and confers chemoresistance [131].

Melanoma, a cutaneous malignancy characterized by high mutational burden and frequent NRAS, BRAF, or NF1 driver mutations [132] secretes EVs enriched in mitochondrial membrane proteins, including MT-CO2 and COX6c. These proteins are detectable in plasma and are significantly elevated in patients with melanoma, ovarian cancer, or breast cancer (C0, Moderate–High quality evidence), establishing a promising diagnostic biomarker platform [133]. Additionally, melanoma cells co-transfer mitochondria and lipid droplets as functional complexes termed lipidosomes. These extracellular lipidosomes concentrate at cell-surface protrusions expressing ITG αVβ3, interacting with matrix proteins prior to release. Expressing surface CD133, lipidosomes activate Wnt/β-catenin signaling in recipient cells to promote cancer stem cell survival, invasiveness, and chemoresistance [134]. While this observation is intriguing, the study remains at the characterization level (C2, Low–Moderate quality) without functional validation of the mitochondrial cargo in recipient cells.

Furthermore, a generalized mechanism observed across multiple tumor types (including TNBC, meningioma, and epithelial pancreatic carcinoma) is evident when tumor cells are subjected to oxidative stress via exposure to carbonylcyanide-3-chlorophenylhydrazone, which induces mitochondrial fragmentation and damage. The tumor cells then bypass lysosomes to extrude these fragments into the extracellular space in a process termed “secretory mitophagy” [99]. Mitochondrial Lon upregulation in cancer cells triggers the release of EVs carrying mtDNA and PD-L1, which suppress T-cell function and promote an immunosuppressive TME, thereby facilitating tumor progression [135]. The levels of mtDNA and PD-L1 found in circulating EVs could serve as potential predictive biomarkers for anti-PD-L1 therapy.

Malignant pleural mesothelioma (MPM), a rare aggressive neoplasm arising from the pleural mesothelium, is frequently diagnosed at an advanced stage and exhibits a dismal therapeutic response [136]. Mesothelioma cell lines produce EVs of various sizes, which can be distinguished by using different centrifugal forces: large-sized EVs isolated at 10,000× *g* (10 K) and at 18,000× *g* (18 K), and then small-sized EVs isolated at 100,000× *g* (100 K). Each of them has been found to carry the mitochondrial enzymes SDHA, FH and IDH1, depressing the activity of oxidative phosphorylation pathway. Additionally, non-mitochondrial cargos in EVs also come into play. For instance, in 10 K EVs, NDUFA13 is located in the mitochondrial inner membrane and acts as a tumor suppresser, PD-L1 inhibits immune response and promotes tumor immune escape, Wnt10A and TCF7L2 proteins activate the Wnt pathway and enhance tumor invasiveness [137]. This study is classified as diagnostic/proteomic characterization (C0, Moderate–High quality) and demonstrates the biomarker potential of mitochondrial-enriched EV subpopulations. However, all findings are derived from in vitro cell lines, and clinical validation in patient samples is required before translational application. The widespread occurrence of these mechanisms across various cancers confirms the importance of EV-mediated mitochondrial trafficking in driving tumor heterogeneity, therapy resistance, and immune modulation, necessitating further research to uncover common therapeutic vulnerabilities (Figure 3, Table 1, Table 2 and Table 3).

**Table 1:** EV-associated intact mitochondria in cancer: evidence from detection to functional effects.

Research Model	Donor Cell	Recipient Cell	EV Subtype	Transported Substance	Isolation and Characterization Methods	Main Effects of Tumor Cells	Targets or Pathways	Evidence Category	Evidence Quality	Key Limitations	References
Triple-negative Breast cancer	Platelets	Breast cancer cells	Platelet-derived extracellular vesicles (PEVs)	Intact mitochondria	Co-culture + FACS sorting (MTG^+^ cells); no EV isolation; confocal/Seahorse/TMRE/qPCR	ATP ↑, OCR ↑, proliferation ↑, colony formation ↑, Rho-0 rescued in uridine/pyruvate-free medium	CD62p ↑, mtDNA repopulation ↑	C2	Low	No EV purification (FACS only); no C2 evidence; no genetic tracing; cannot distinguish EV-encapsulated from free mitochondria; in vitro only; no causal validation	Cereceda L et al. (2024) [91]
Breast cancer	Platelets	Breast cancer cells	mitoMPs	Functional intact mitochondria	/	OCR ↑, ATP ↑, migration ↑, invasion ↑	OXPHOS-dependent	C2	Low	No EV isolation methods; no C2 evidence (no detergent/DNase/immunogold); no genetic tracing/uptake imaging; functional effects observed but EV-mediated delivery not established; in vitro only; no causal validation	Veilleux V et al. (2024) [92]
Triple-negative breast cancer	Chemoresistant TNBC cells	Chemosensitive TNBC cells	Exosomes	Mitochondria with mutant mtDNA (mtND4 mutations)	/	Chemoresistance ↑	mtDNA levels ↑, Mutations ↑ in mtND4 gene	C2	Low–Moderate	No C2 evidence; no genetic tracing; in vitro only; no causal validation	Abad E et al. (2022) [97]
Chronic lymphocytic leukemia (CLL)	Platelets	CLL cells	Platelet-derived microparticles (PMPs)	Functional intact mitochondria	Differential centrifugation (17,800× *g*, 90 min); flow cytometry (CD41^+^, MTDR^+^, PKH67^+^); freshly isolated (never frozen)	↑ proliferation, ↑ mtDNA copy number, ↑ OCR, ↑ ATP, ↑ ROS; ↑ migration, ↑ invasion; ↑ chemoresistance (Cytarabine, Venetoclax, Plumbagin)	PMP-recipient CLL cells ↑ in G2/M, NF-ĸB pathway ↑, HIF-1α pathway ↑, MCL1 ↑	C2	Low–Moderate	No density gradient to exclude free mitochondria; no C2 evidence; no genetic tracing; in vitro only; no causal validation	Gharib E et al. (2023) [110]
Gastric cancer	Mesenchymal stromal cells (MSCs)	GC cells	Microvesicles	Intact mitochondria	Ultracentrifugation; AFM for matrix stiffness; RhoA/ROCK1 pathway inhibition; confocal/flow cytometry (MitoTracker)	↑ mitochondrial content (biosynthesis-independent); mitochondrial function repair; ↓ mitophagy; ↑ oxaliplatin resistance	Oxaliplatin and RhoA/ROCK1 pathway ↓, ECM ↑, MVs ↑	C2	Moderate	No density gradient; no detergent protection; no genetic tracing; MV vs. free mitochondria unclear; in vitro + in vivo; no causal validation	He X et al. (2024) [125]
Melanoma	Melanoma cells (FEMX-I, metastatic)	Surrounding tissues	Large CD133^+^ EVs (lipidosomes, 2–6 µm)	Intact mitochondria + lipid droplets	Differential centrifugation (200,000× *g*); SEM/TEM; confocal live-cell imaging; CD133 silencing; BODIPY/MitoTracker staining	Metabolic activity ↑, Metastasis ↑, Aggressiveness ↑, Resistance of cells ↑	ITGαVβ3 ↑ with ECM protein, CD133 ↑, Wnt/β-catenin pathway ↑	C2	Low–Moderate	Structural characterization only; no functional validation; no detergent protection; no genetic tracing; in vitro only; no causal validation	Karbanová J et al. (2024) [134]

Abbreviations: C1–C4, evidence category levels. C1 (Detection): Cargo detected in EV-enriched preparations; no demonstration of intravesicular localization, contaminant exclusion, or intercellular delivery. C2 (Localization): Intravesicular localization and/or contaminant exclusion demonstrated (e.g., detergent protection, nuclease protection, immunogold, EV positive/negative markers). Intercellular delivery alone, without cargo localization, is not sufficient for C2. C3 (Functional Effect, EV-Mediated): Cargo localization (C2) AND evidence of intercellular delivery AND functional effects in recipient cells (e.g., OCR, ATP, migration, chemoresistance). C4 (Causal Validation): C3 criteria AND cargo-specific loss-of-function or rescue experiments (KO, siRNA, pharmacological inhibition, complementation) to establish that the observed phenotype is directly attributable to the transferred mitochondrial cargo. C0 is used for diagnostic studies with no functional validation. Evidence Quality: High, meets all of advanced purification + intravesicular localization + functional effect + causal validation; Moderate, meets some but lacks causal validation or has minor methodological gaps; Low, only conventional ultracentrifugation/WB/PCR, no localization or contaminant exclusion, or no EV purification. Arrows: Symbols ↑ and ↓ indicate an increase or decrease, respectively, in the parameter or pathway as reported in the original study. These arrows denote directional changes observed under the experimental conditions of the cited study and should not be interpreted as universal or validated causal effects unless explicitly stated.

**Table 2:** Detection and signaling of EV-encoded mitochondrial components (mtDNA/mtRNA/Proteins/Lipids/Fragments).

Research Model	Donor Cell	Recipient Cell	EV Subtype	Transported Substance	Isolation and Characterization Methods	Main Effects of Tumor Cells	Targets or Pathways	Evidence Category	Evidence Quality	Key Limitations	References
Breast cancer	Breast cancer cells	Glutamine-starved cancer cells	Undefined	mtDNA	Differential ultracentrifugation + sucrose density gradient; NTA; WB (CD63^+^, CD9^+^, Flotillin^+^); CD63 immunogold TEM; DNase-conjugated beads; proteinase K; PINK1 CRISPR/Cas9 KO + rescue; TLR9 siRNA/antagonist	Migration ↑, invasion ↑	Mitochondrial damage, PINK1 ↑, mGluR3 ↑, Rab27-dependent exocytosis of EVs ↑, TLR9-dependent pro-invasive endosomal trafficking ↑	C4	Moderate–High	C2 present (DNase, immunogold, density gradient); genetic tracing via PINK1 KO/rescue; in vitro + in vivo; limitation: no density gradient sub-fractionation; limited in vivo data	Rabas N et al. (2021) [95]
Prostate cancer	PC3 cells	PC3 cells	Undefined	Mitochondrial proteins	Affinity kit (Qiagen ExoEasy); NTA (ZetaView); TEM; Jess automated capillary WB; EV uptake (exo-glow + confocal)	Survival ↑, Anti-apoptotic ↑ after radiotherapy	H_2_O_2_ ↑, Mitochondrial respiratory impairment ↑, EVs release	C2	Moderate	No density gradient; no detergent protection; no DNase; affinity kit may co-purify contaminants; no genetic tracing; in vitro only; no causal validation	Miller CE et al. (2022) [102]
Prostate cancer	Senescent tumor cells	PMN-MDSCs	Undefined	mtDNA	Density gradient/ultracentrifugation (STAR Methods); VDAC inhibition	Immunosuppression of PMN-MDSCs ↑, Progression ↑	mtDNA release ↑ via VDAC, cGAS-STING-NF-kB pathway ↑	C2	Moderate	No detergent protection; no DNase; no genetic tracing; causal via VDAC inhibition (indirect); in vitro + in vivo; no cargo-specific KO/rescue	Lai P et al. (2025) [103]
Lung cancer	High-metastatic A11 cells (ND6 G13997A mutant mtDNA)	Low-metastatic P29 cells; CAFs; macrophages; T cells	S-EVs and L-EVs	mtDNA, Degenerated mitochondrial fragments	Differential ultracentrifugation (15,000× *g* for L-EVs; 110,000× *g* for S-EVs); 0.22 µm filtration; NTA; WB (Annexin A1^+^, CD9^+^, CD63^+^, CD81^+^, LC3-II^+^); TEM (negative-stain + cryo); PCR (HVR, ND1, COI, ND4, ND6); PCR-RFLP (G13997A); GW4869 + tipifarnib	Bidirectional transfer between high- and low-metastatic cells; transfer to CAFs/macrophages/T cells; mtDNA transfer to ρ^0^ cells	ND6 G13997A → metastasis enhancement; autophagy-related S-EVs (LC3-II^+^)	C3	Moderate–High	C2 present (EV isolation, TEM, WB, PCR); no detergent protection; DNase not described; causal via GW4869 (indirect); in vitro + in vivo; no mtDNA-specific KO/rescue	Takenaga K et al. (2021) [115]
Head and neck squamous cell carcinoma (HNSCC)	HNSCC cells (HSC3, MOC1)	Bone marrow-derived macrophages (BMDMs)	sEVs	Mitochondria-related proteins (cargo altered by HRS depletion under hypoxia)	Differential centrifugation; NTA; TEM; WB (CD63^+^, TSG101^+^, Alix^+^); HRS KD (shRNA); HIF-1α KD; mass spectrometry; ChIP-qPCR (HIF-1α binds HRS promoter); spatial transcriptomics	Immunosuppression ↑, Progression ↑, Metastasis ↑	HIF-1 ↑, HRS ↑, ESCRT ↑, MVBs ↑	C3	Moderate–High	C2 present (NTA, TEM, WB, HRS/HIF-1α KD, ChIP); no detergent protection; no DNase; causal via HRS/HIF-1α KD (indirect); in vitro + patient samples; no cargo-specific KO/rescue	Wang Y et al. (2025) [118]
Head and neck squamous cell carcinoma (HNSCC)	FaDu cells	None	Undefined	mtRNAs	MagCapture Exosome Isolation Kit (PS Ver. 2); NTA (NanoSight); TEM (negative-stain + cryo); WB (TSG101, CD9, Flotillin-1, Annexin V, Caveolin-1); RNA-seq; qRT-PCR in patient serum	EV mtRNA cargo characterization, altered mtRNA abundance in EVs under starvation/autophagy induction, mtRNAs detectable in HNSCC patient serum EVs	Autophagy induction (starvation/BEZ) → mitochondrial fragmentation → TSG101 localization on mitochondrial membrane (observed), altered mtRNA abundance in EVs	C1	Moderate	No recipient cell; no uptake assay; no genetic tracing; no functional effects assessed (proliferation/metastasis/invasion not evaluated); mechanistic pathway inferred from donor-cell observations only; in vitro + human serum; no causal validation	Bugajova M et al. (2024) [117]
Liver cancer	Liver cancer cells	HUVECs, HepG2 cells	MDVs (distinct from classical EVs)	Mitochondrial proteins, mtDNA	Differential ultracentrifugation (100,000× *g*); TEM; nanoflow cytometry (nanoFCM); mass spectrometry; WB (CD63^+^, TSG101^+^); NOC (microtubule inhibitor) to block MDV formation	Angiogenesis ↑, invasion ↑, tumor spheroid growth, in vivo tumor growth and metastasis ↑	HSP90AA1-HCFC1 → TFEB transcription → LC3-mediated mitophagy; HSP90 inhibition → ↓ TFEB → ↓ LC3 → ↑ MDVs → ↑ EV release of mitochondrial components → metastasis	C3	Moderate-High	C2 present (TEM, nanoFCM, WB, mass spec); no detergent protection; no DNase; causal via NOC/HSP90 inhibition (indirect); in vitro + in vivo; no cargo-specific KO/rescue	Liu L et al. (2025) [122]
Colonic cancer	Colonic cancer cells	Adjacent colonic epithelial cells (CECs)	Undefined	Circular mtDNA	Differential ultracentrifugation (120,000× *g*); sucrose density gradient; TEM; NTA; WB (CD63^+^, ALIX^+^, CD9^+^, TSG101^+^; Calnexin^−^, GM130^−^); DNase treatment; long-range PCR (intact mtDNA); Rel A ChIP; TFAM KD	Mitochondrial respiration (OCR) ↑, ↑ ROS, tumor progression ↑	Mitochondrial respiratory chain ↑, ROS-driven RelA nuclear translocation ↑, TGF1 expression ↑, TGF/Smad pathway↑	C4	High	C2 present (sucrose gradient, TEM, NTA, WB, DNase, ChIP); genetic tracing via TFAM KD; in vitro + in vivo; limitation: TFAM KD (indirect mtDNA manipulation); no direct mtDNA-specific KO	Guan B et al. (2024) [123]
Ovarian cancer	Ovarian cancer cells (A2780 CIS, cisplatin-resistant)	Ovarian cancer cells (A2780, cisplatin-sensitive)	MDVs (distinct from classical EVs)	mtDNA + mitochondrial proteins	Ultracentrifugation/immunoisolation; protease protection assay; TEM; NTA; WB (Alix^+^, TSG101^+^, CD63^+^, CD9^+^, CD81^+^; RPS6^−^); DNase treatment; PCR (MT-ND6, MT-RNR1, MT-COX1/2, MT-ND3/ND4L); RAB7 OE/KD; GW4869	MDV secretion ↑, Mitophagy ↓, CDDP-resistance ↑	RAB7 ↓, RAB7 ↑ in EVs, TFEB-mTOR pathway ↓	C3	Moderate–High	C2 present (protease protection, DNase, TEM, NTA, WB); genetic tracing via RAB7 OE/KD; causal via GW4869/RAB7 modulation (indirect); in vitro only; no mtDNA-specific KO/rescue	Gagliardi S et al. (2024) [131]
Oral squamous cell carcinoma	OSCC cells (HSC3, OEC-M1)	Macrophages (RAW264.7, THP1); T cells	Undefined	Oxidized mtDNA + PD-L1	Differential ultracentrifugation (120,000× *g*); TEM; WB (CD63^+^, TSG101^+^, HSP70^+^); long-range PCR (mtDNA integrity); 8-OHdG ELISA (oxidized mtDNA); TLR9 activation assay; PD-L1 antibody	Progression ↑, immunity attenuation	Lon → oxidized mtDNA → cGAS-STING → IFN → PD-L1 ↑; EV-mediated paracrine immunosuppression	C3	Moderate–High	C2 present (TEM, WB, long-range PCR, 8-OHdG ELISA); no detergent protection; no DNase; causal via Lon modulation (indirect); in vitro + in vivo + patient samples; no mtDNA-specific KO/rescue	Cheng AN et al. (2020) [135]
Breast cancer meningioma, epithelial pancreatic carcinoma	4T1 cell, IOMM-Lee cell, PANC1 cell, Murine undifferentiated macrophages	None	Undefined	Damaged mitochondrial fragments	Differential centrifugation (500 *g* → 2000 *g* → 10,000 *g* → 100,000 *g*); NTA (ZetaView); WB (CD81^+^, Calnexin^−^, PINK1^+^); CCCP + Baf A1 treatment	ATP ↑, oxidative damage ↓, Survival ↑, progression ↑, apoptosis ↓	Secretory mitophagy ↑, PINK1 ↑, EVs ↑	C3	Moderate	C2 present (NTA, WB with CD81^+^/Calnexin^−^); no detergent protection; no genetic tracing; no uptake assay (donor-cell characterization only); causal via Baf A1 (indirect); in vitro only; no cargo-specific KO/rescue	Gade PV et al. (2025) [99]

Abbreviations: C1–C4, evidence category levels. C1 (Detection): Cargo detected in EV-enriched preparations; no demonstration of intravesicular localization, contaminant exclusion, or intercellular delivery. C2 (Localization): Intravesicular localization and/or contaminant exclusion demonstrated (e.g., detergent protection, nuclease protection, immunogold, EV positive/negative markers). Intercellular delivery alone, without cargo localization, is not sufficient for C2. C3 (Functional Effect, EV-Mediated): Cargo localization (C2) AND evidence of intercellular delivery AND functional effects in recipient cells (e.g., OCR, ATP, migration, chemoresistance). C4 (Causal Validation): C3 criteria AND cargo-specific loss-of-function or rescue experiments (KO, siRNA, pharmacological inhibition, complementation) to establish that the observed phenotype is directly attributable to the transferred mitochondrial cargo. C0 is used for diagnostic studies with no functional validation. Evidence Quality: High, meets all of advanced purification + intravesicular localization + functional effect + causal validation; Moderate, meets some but lacks causal validation or has minor methodological gaps; Low, only conventional ultracentrifugation/WB/PCR, no localization or contaminant exclusion, or no EV purification. Arrows: Symbols ↑ and ↓ indicate an increase or decrease, respectively, in the parameter or pathway as reported in the original study. These arrows denote directional changes observed under the experimental conditions of the cited study and should not be interpreted as universal or validated causal effects unless explicitly stated.

**Table 3:** Engineered EVs and translational diagnostic/therapeutic applications.

Research Model	Donor Cell	Recipient Cell	EV Subtype	Transported Substance	Isolation and Characterization Methods	Main Effects of Tumor Cells	Targets or Pathways	Evidence Category	Evidence Quality	Key Limitations	References
Breast cancer	Breast cancer cells	None	Undefined	Mitochondrially encoded mRNAs	SEC (Sepharose CL2B) + concentration; NTA (NanoSight); TEM; WB (TSG101^+^/Calnexin^−^/CD63^+^); proteinase K + RNase A treatment; RNA-seq	Diagnostic: AUC 0.902 (LOOCV) distinguishing BC from healthy controls; prediction of ER/HER2 status	/	C0	Moderate–High	Diagnostic only; no functional experiments; no genetic tracing; no causal validation; EV subtypes not defined; limited sample size; requires independent cohort validation	Zayakin P et al. (2023) [96]
Pancreatic cancer	Dendritic cells	Dendritic cells, T cells/NK cells	DC-derived microvesicles	mtDNA	cNP loading with cell lysate → DC uptake → release of cNP^cancer cell^@MV^DC^; SEM; NTA; proteomics/phosphoproteomics; in vivo imaging	Antitumor immunity ↑, tumor growth ↓	cNPs enrich mtDNA, cGAS-STING pathway ↑	C3	Moderate–High	Engineered therapeutic application; C2 present (SEM, NTA, in vivo imaging); no mtDNA-specific KO/rescue; clinical translation needs further validation; in vitro + in vivo	Shang L et al. (2024) [129]
Pancreatic cancer	Pancreatic cancer cells	None	Undefined	mtDNA	Immunoprecipitation (IP) + qPCR (EvIPqPCR); DNA isolation-free; duplexing probes; mtDNA/nDNA ratio detection	Diagnostic: discriminating healthy controls, pancreatitis, and pancreatic cancer	/	C0	Moderate	Diagnostic only; no functional experiments; no genetic tracing; no causal validation; limited sample size; prospective validation needed	Rasuleva K et al. (2023) [128]
Pancreatic ductal adenocarcinoma	PDAC cells	None	Undefined	mtDNA and cardiolipin	SEC; protein quantification; REPLI-g mtDNA enrichment; NGS (Variant Pro, full mitochondrial genome); qPCR (ND1/GAPDH); cardiolipin assay	Diagnostic: ↑ mtDNA mutation frequency in PDAC-EVs; ↑ mtDNA copy number (14-fold); ↑ cardiolipin	/	C0	Moderate–High	Diagnostic only; no functional experiments (no uptake/migration/invasion/immune effects); no detergent/DNase; sample size limited (*n* = 20 PDAC); independent cohort validation needed	Vikramdeo KS et al. (2022) [130]
Melanoma	Melanoma metastatic tissue/cell lines (MML1, etc.)	None	Undefined	Mitochondrial membrane proteins, active mitochondrial enzymes	Tissue digestion + differential centrifugation (16,500× *g* + 110,000× *g*); iodixanol density gradient; membrane isolation (carbonate pH 12); TEM; WB (CD81^+^, MT-CO2^+^); LC-MS/MS; ELISA (direct/sandwich); immunomagnetic capture (MT-CO2^+^ EVs)	Detection/diagnostic: melanoma tissue EVs enriched in mitochondrial membrane proteins; MT-CO2/COX6c elevated in plasma of melanoma/ovarian/breast cancer patients	/	C0	Moderate–High	Diagnostic only; no functional experiments; no genetic tracing; no causal validation; EV subtypes not separated (large + small combined); sample size limited; clinical validation needed	Jang SC et al. (2019) [133]
Malignant pleural mesothelioma (MPM)	Mesothelioma cell lines	Immune/tumor/stromal cells	10 K, 18 K, 100 K EVs	Mitochondrial components (10 K EVs enriched); Mesothelin (100 K EVs); PD-L1/PD-L2 (10 K EVs)	Differential centrifugation (10,000× *g*, 18,000× *g*, 100,000× *g* + sucrose-D_2_O cushion); NTA (NanoSight); TEM; flow cytometry (Annexin V); WB; LC-MS/MS (4054 proteins); IPA analysis	Diagnostic/biomarker: distinct EV subtypes with unique cancer-specific proteomes; 46 novel mesothelioma-associated proteins	/	C0	Moderate–High	Diagnostic/proteomic only; no functional experiments; no genetic tracing; no causal validation; in vitro cell line-derived; clinical validation in patients needed	Ahmadzada T et al. (2023) [137]

Abbreviations: C1–C4, evidence category levels. C1 (Detection): Cargo detected in EV-enriched preparations; no demonstration of intravesicular localization, contaminant exclusion, or intercellular delivery. C2 (Localization): Intravesicular localization and/or contaminant exclusion demonstrated (e.g., detergent protection, nuclease protection, immunogold, EV positive/negative markers). Intercellular delivery alone, without cargo localization, is not sufficient for C2. C3 (Functional Effect, EV-Mediated): Cargo localization (C2) AND evidence of intercellular delivery AND functional effects in recipient cells (e.g., OCR, ATP, migration, chemoresistance). C4 (Causal Validation): C3 criteria AND cargo-specific loss-of-function or rescue experiments (KO, siRNA, pharmacological inhibition, complementation) to establish that the observed phenotype is directly attributable to the transferred mitochondrial cargo. C0 is used for diagnostic studies with no functional validation. Evidence Quality: High, meets all of advanced purification + intravesicular localization + functional effect + causal validation; Moderate, meets some but lacks causal validation or has minor methodological gaps; Low, only conventional ultracentrifugation/WB/PCR, no localization or contaminant exclusion, or no EV purification. Arrows: Symbols ↑ and ↓ indicate an increase or decrease, respectively, in the parameter or pathway as reported in the original study. These arrows denote directional changes observed under the experimental conditions of the cited study and should not be interpreted as universal or validated causal effects unless explicitly stated.

### 5.8. Critical Appraisal of the Evidence

While the studies discussed above collectively demonstrate the widespread nature of intercellular mitochondrial transfer, it is imperative to critically distinguish between studies that provide unequivocal evidence for the functional transfer of intact, respiration-competent mitochondria and those that merely report the detection of mitochondrial components within EV preparations. It is important to note that among the studies summarized, only a subset provide evidence for the transfer of functional intact mitochondria (defined as organelles capable of respiration and ATP production in recipient cells). These studies, such as platelet-derived mitochondrial transfer to breast cancer cells and CLL cells, as well as MSC-derived mitochondrial transfer to gastric cancer cells, include direct functional validation (e.g., restoration of OCR, ATP production, or rescue of mtDNA-depleted Rho-0 cells). By contrast, studies demonstrating functional transfer of mitochondrial nucleic acids (mtDNA or mtRNA), such as EV-mediated mtDNA transfer in colonic cancer where internalized mtDNA activates the mitochondrial respiratory chain and downstream TGFβ/Smad signaling, represent a mechanistically distinct category. They demonstrate functional consequences of nucleic acid delivery rather than transfer of intact, respiration-competent organelles, and thus should not be conflated with bona fide intact mitochondrial transfer. In contrast, the majority of studies detect mitochondrial components (mtDNA, mtRNA, or mitochondrial proteins) in EVs without demonstrating functional activity of transferred mitochondria. These findings support a role for EVs in delivering mitochondrial-derived signals (e.g., DAMPs) or metabolic information, but do not constitute evidence of functional mitochondrial organelle transfer. This distinction is critical for interpreting the biological significance and therapeutic potential of EV-mediated mitochondrial communication.

## 6. Therapeutic Implications of Mitochondrial Transfer

EVs have been extensively used as emerging delivery vectors for diverse therapeutic cargos. The therapeutic applications of EV–mitochondria interactions in cancer can be broadly categorized into two conceptually distinct approaches. The first category includes studies that utilize EVs as delivery vehicles to transport exogenous agents (e.g., drugs, photosensitizers, glycolysis inhibitors, or other antitumor compounds) specifically to mitochondria in cancer cells. These approaches represent EV-mediated drug delivery to mitochondria, rather than EV-mediated transfer of mitochondria or mitochondrial components. The second category, discussed in preceding sections, involves the intercellular transfer of endogenous mitochondrial cargo (e.g., mtDNA, mitochondrial proteins, or intact mitochondria) via EVs, which represents a biological communication mechanism rather than a therapeutic engineering strategy. Notably, studies falling into the first category should not be conflated with evidence of endogenous mitochondrial transfer, as they address fundamentally different biological and translational questions. For instance, migrasomes selectively expel damaged mitochondria via KIF5B-MYO19-dependent mitocytosis, and they act as positioning signals to promote invasion, angiogenesis, and immunosuppression [138]. Large EVs derived from MSCs mediate functional mitochondrial transfer to cardiomyocytes, restoring mitochondrial biogenesis, ATP production, and contractility while attenuating doxorubicin-induced apoptosis, revealing a regenerative mechanism independent of direct cell engraftment [139]. Small EVs engineered to encapsulate a glutathione-depleting agent effectively overcome drug efflux mediated by P-glycoprotein, restoring chemosensitivity of multidrug-resistant ovarian cancer by inducing oxidative stress, mitochondrial dysfunction, and ATP depletion, leading to enhanced potency against resistant tumors [140]. In contrast to the native EV-mediated processes described above, the following studies utilize engineered EVs as therapeutic vehicles for mitochondrial targeting. Nguyen Cao TG demonstrates that engineered EVs encapsulating a sonosensitizer and a pro-oxidant drug can effectively target mitochondria, amplify reactive oxygen species generation, and achieve potent anticancer activity in both cell-based and animal models [141]. EVs produced by brain endothelial cells enable targeted delivery of a mitochondria-localizing photosensitizer across the blood–brain barrier, achieving enhanced photodynamic therapy efficacy and significant tumor growth inhibition in orthotopic glioblastoma models without systemic toxicity [142]. Similarly, bioreducible exosomes enable tumor-specific co-delivery of a mitochondria-targeting sonosensitizer and glycolysis inhibitor, triggering glutathione-responsive drug release and ultrasound-activated mitochondrial damage to potentiate sonodynamic therapy [143]. Collectively, a deeper understanding of the regulatory networks governing mitochondrial transfer not only reshapes our view of tumor malignancy but also holds promise as a potential breakthrough for precision treatment of tumors.

## 7. Conclusions and Perspectives

EV-mediated transfer of mitochondria and their components has become a biologically important mechanism of intercellular communication in TME. This phenomenon has been observed across diverse malignancies, including solid tumors such as breast, prostate, colon, gastric, and ovarian cancers, as well as hematological malignancies such as CLL and AML. Functional consequences reported in individual studies include enhanced metabolic capacity, increased proliferative and invasive potential, resistance to chemotherapy, and modulation of the immune microenvironment. However, as reflected in the evidence grading summarized in Table 1, Table 2 and Table 3, the strength of evidence varies substantially across cancer types. The majority of studies remain at C1 or C3 level, with only a minority achieving C4, due to the persistence of critical methodological gaps, including inadequate exclusion of free mitochondria and non-EV contaminants, insufficient confirmation of intravesicular cargo localization, and limited causal validation through cargo-specific loss-of-function or rescue experiments. We emphasize that the field is still in the formative stage. Many observations await rigorous validation with appropriate controls, and the pathophysiological relevance of EV-mediated mitochondrial transfer in cancer remains to be fully elucidated. Therefore, future research should prioritize standardized methodology for EV characterization, contaminant exclusion, and causal validation, as well as the development of in vivo models that accurately recapitulate this transfer in physiological settings. Understanding the full scope and significance of this intercellular communication pathway will require coordinated efforts to move beyond descriptive observations toward mechanistic and functional validation.

### 7.1. Mechanistic Comparison in EV-Mediated Communication

The mechanisms by which EVs mediate mitochondrial communication vary substantially depending on the transported cargo. We propose a framework to compare these mechanisms across five steps: (A) cargo selection, (B) EV biogenesis and release, (C) recipient-cell uptake, (D) intracellular fate, and (E) functional integration. Importantly, the observations summarized below are drawn from specific model systems and should not be generalized as universal principles across all cell types or cancer contexts.

Mechanistic insights into cargo sorting have emerged primarily from individual model systems. MtDNA packaging into EVs has been mechanistically linked to PINK1 in the breast cancer model [95] via a Rab27-dependent mechanism, as well as to ESCRT-dependent pathways via HRS in HNSCC cells under hypoxia [118], where HRS depletion alters the packaging of mitochondria-related proteins into sEVs. VDAC-dependent mtDNA release has been described in therapy-induced senescent prostate cancer cells [103], where mtDNA is released via VDAC channels and subsequently encapsulated in EVs. We can see mtRNA abundance in EVs increase upon autophagy induction in HNSCC cells [117], though the precise packaging mechanism remains incompletely defined. In addition, mitochondrial proteins are sorted into MDV-derived EVs under HSP90 inhibition in hepatocellular carcinoma [122], a process linked to decreased TFEB transcription and reduced LC3-mediated mitophagy, as well as via HRS-dependent mechanisms under hypoxia in HNSCC [118]. Intact mitochondrial packaging into EVs has been associated with RhoA/ROCK1-mediated cytoskeletal dynamics in the gastric cancer model [125], where matrix stiffness promotes MSC-to-cancer cell mitochondrial transfer via microvesicles.

Recipient-cell uptake of EV-associated mitochondrial cargo is highly context-dependent. In the melanoma model [134], large CD133^+^ EVs (2–6 µm) containing mitochondria and lipid droplets were released during cell division and taken up by recipient cells. In the ovarian cancer model [131], MDVs were taken up by recipient cells and localized to the mitochondria. In the colon cancer model [123], tumor-derived EVs were taken up by adjacent colonic epithelial cells. In the prostate cancer model [102], radiation-derived EVs were internalized by recipient cells as confirmed by exo-glow labeling. Following uptake, mtDNA-containing EVs activated endosomal TLR9 in the breast cancer model [95] or cytosolic cGAS-STING in the prostate cancer senescence model [103] and the oral cancer model [135].

The functional outcomes similarly defy simple categorization. Intact mitochondrial transfer has been associated with restoration of OXPHOS and ATP production in recipient cells in the breast cancer models [91,92], the CLL model [110], and the gastric cancer model [125]. MtDNA delivery can exert metabolic effects through mitochondrial gene expression or genetic complementation, for example, restoration of respiration in ρ^0^ cells [115] or enhanced OCR in adjacent colonic epithelial cells [123], in addition to activating innate immune signaling via cGAS-STING [103,135] or TLR9 [95]. Mitochondrial proteins may retain enzymatic or signaling functions, as demonstrated by ATP synthase activity in melanoma tissue-derived EVs [133] and by pro-angiogenic and pro-metastatic effects of MDV-derived mitochondrial proteins in hepatocellular carcinoma [122]. Immune responses elicited by EVs are driven primarily by vesicular mtDNA cargo. In prostate cancer senescence models, mtDNA-loaded EVs enhance the immunosuppressive capacity of PMN-MDSCs via cGAS–STING-NF-κB activation [103]. Similarly, in oral squamous cell carcinoma models, EV-encapsulated oxidized mtDNA and PD-L1 inhibit T-cell function through cGAS–STING and type I interferon signaling [135]. Thus, the biological impact of EV-mediated mitochondrial transfer forms a continuous functional spectrum shaped by cargo identity, delivery route, and recipient microenvironment, rather than a strict binary distinction between metabolic and immune outcomes.

### 7.2. Challenges and Future Directions

Beyond the biological mechanisms discussed, the broader field of EV-based therapeutics has also advanced into clinical testing. For example, CDK-004 (exoASO-STAT6), an engineered exosome platform delivering a STAT6 antisense oligonucleotide to repolarize tumor-associated macrophages, entered a Phase I clinical trial in patients with advanced hepatocellular carcinoma (HCC) or liver metastases from gastric or colorectal cancer (NCT05375604). However, this trial was terminated early, and it is important to emphasize that this represents a general example of an engineered EV drug-delivery platform rather than evidence that mitochondrial transfer-based therapy has entered the clinic [144]. Exosomes serve as biocompatible vehicles that safely encapsulate diverse therapeutics, offering low toxicity, favorable pharmacokinetics, and inherent organotropism. Over the past decade, innovations including microfluidics, nanoporation, fusogenic hybrids, and genetic engineering have substantially enhanced their loading efficiency, production scalability, and pharmacokinetic performance [145]. However, the successful translation of mitochondrial transfer-based therapies faces substantial hurdles. These include pronounced EV heterogeneity (e.g., size, density, and surface protein composition), potential contamination that may confound functional readouts by protein/lipoprotein, mitochondria or mitochondrial fragments with traditional EV isolation methods [146], unpredictable biodistribution profiles upon in vivo administration, and unresolved safety concerns regarding immunogenicity and off-target effects. Furthermore, robust biomarker validation for patient stratification and precise therapeutic targeting remain major challenges. Crucially, the therapeutic efficacy of EV-mediated mitochondrial delivery is not merely determined by the delivery vehicle itself; it is intimately governed by the donor cell origin and its stress state (e.g., hypoxic or inflammatory conditions), which dictate the composition of the mitochondrial cargo. Upon recipient-cell uptake, these distinct cargo types can differentially engage host immune surveillance pathways, particularly the cGAS–STING axis and TLR9, thereby either potentiating antitumor immunity or inadvertently fostering an immunosuppressive milieu. A deep understanding of this donor–cargo–immune axis is paramount for guiding rational engineering strategies. These tailored approaches hold promise for translating mitochondrial cargo transfer into specific diagnostic applications and therapeutic interventions.

The collective findings discussed in this review present a fact that intact mitochondria and mitochondria-derived components function as intercellular signaling entities in TME. Nevertheless, it is critical to recognize that the available evidence remains predominantly preclinical and preliminary, with the majority of data derived from in vitro co-culture systems and xenograft mouse models, rather than from clinical settings. This inherent uncertainty, particularly regarding the physiological relevance and stoichiometric efficiency of mitochondrial transfer, must be explicitly acknowledged as a significant limitation of the current literature.

A central methodological challenge in this field is the difficulty of unequivocally distinguishing EV-encapsulated mitochondria or mitochondrial components from free mitochondria, mitochondrial fragments, and other non-EV contaminants. Intact mitochondria, mitochondrial fragments, large EVs, apoptotic bodies, protein aggregates, and lipoproteins can overlap considerably in size, density, and sedimentation behavior. Consequently, conventional differential or ultracentrifugation, transmission electron microscopy, nanoparticle tracking analysis, Western blotting for mitochondrial markers, or MitoTracker staining does not by itself demonstrate intact mitochondria are enclosed within EVs. To rigorously establish EV-mediated mitochondrial cargo transfer, several orthogonal lines of evidence are required. First, EV preparations should be separated from free mitochondria using density gradient centrifugation or size-exclusion chromatography, combined with appropriate EV positive markers and negative markers (e.g., calnexin, GM130, apolipoproteins). Second, intravesicular localization of mitochondrial cargo should be confirmed by protease or nuclease protection assays, in which resistance to enzymatic digestion is observed in intact EVs but lost upon detergent permeabilization. Third, donor-specific genetic tracing (e.g., mitochondrial-tagged GFP or photoconvertible fluorescent proteins) should be used to unambiguously demonstrate intercellular transfer and distinguish it from passive uptake of free mitochondria. Fourth, for studies claiming transfer of functional intact mitochondria, additional validation should include measurements of mitochondrial membrane potential (e.g., TMRE or JC-1 staining), respiratory activity (e.g., Seahorse OCR), and, ideally, demonstration of integration of donor mitochondria into the recipient cell mitochondrial network. In the present review, we applied these criteria to assess the available literature. A distinguishing feature of this review is the systematic application of an evidence-grading framework (C1–C4) to the available literature. It provides readers with a clear assessment of the strength of evidence for each cancer type, which is not readily available from previous narrative reviews, identifing clear methodological priorities for future research. By making these gaps explicit, we hope to guide experimental design in future studies and to accelerate the translational potential of this field. Among the studies surveyed, only a minority fulfilled the stringent requirements for causal validation (C4), while the majority of studies remained at the detection (C1) or functional effect (C3) level without full exclusion of contaminants or formal causal proof. We explicitly highlight these methodological limitations in Table 1, Table 2 and Table 3 and in the cancer-specific discussions below.

Moving forward, several critical challenges and opportunities remain to be addressed. Future research must prioritize elucidating the precise molecular signals that govern the selective packaging of mitochondria into EVs, the transfer of mitochondrial cargo, and the mechanisms dictating their uptake by recipient cells. Investigations with standardized in vivo lineage tracing, rigorous exclusion of non-specific uptake, and functional recovery assays are urgently needed to transition this field from descriptive observations to mechanistic understanding. Deciphering the ultimate intracellular fate and functional integration of these transferred components within recipient cells represents a crucial area of investigation. From a translational perspective, therapeutic interventions targeting EV-mediated mitochondrial transfer hold potential clinical promise. In particular, engineered EVs designed to package and deliver functional mitochondrial cargo represent a versatile strategy for restoring metabolic homeostasis in treating diseases. However, the successful clinical translation of such engineered platforms will require rigorous optimization of loading efficiency, targeting specificity, and in vivo safety profiles. Future therapeutic strategies could involve inhibiting the release or uptake of mitochondrial EVs, or exploiting them as robust bioindicators for early detection, prognostic assessment, and monitoring therapeutic responses. Ultimately, unraveling the complexities of this intercellular mitochondrial communication will not only deepen our understanding of tumor heterogeneity and adaptation, but also lay the foundation for groundbreaking combination therapies aimed at disrupting cancer progression and therapeutic resistance.

## Data Availability

No new data were created or analyzed in this study. Data sharing is not applicable to this article.

## Abbreviations

The following abbreviations are used in this manuscript:

AML	Acute Myeloid Leukemia
AR	Androgen Receptor
BM	Bone Marrow
CECs	Colonic Epithelial Cells
CLL	Chronic Lymphocytic Leukemia
ECM	Extracellular Matrix
ERM	Ezrin/Radixin/Moesin
ESCRT	Endosomal Sorting Complexes Required for Transport
EV	Extracellular Vesicles
GC	Gastric Cancer
GJIC	Gap Junction Intercellular Communication
HNSCC	Head and Neck Squamous Cell Carcinoma
HS	Heparan Sulfate
MDVs	Mitochondrial-derived Vesicles
MSCs	Mesenchymal Stem Cells
MVs	Microvesicles
NSCLC	Non-small Cell Lung Cancer
OCR	O_2_ Consumption Rate
OXPHOS	Oxidative Phosphorylation
PMVs	Platelet-derived Microvesicles
ROS	Reactive Oxygen Species
TEVs	Tumor-derived EVs
TFEB	Transcription Factor EB
TME	Tumor Microenvironment
TMs	Tumor Microtubes
TNBC	Triple-Negative Breast Cancer
TNTs	Tunneling Nanotubes
VDAC	Voltage-Dependent Anion Channel
VEGF	Vascular Endothelial Growth Factor
